# HIV nucleocapsid proteins as targets for novel 1,2-benzisothiazol-3(2H)-one benzenesulfonamides: synthesis and antiretroviral activity

**DOI:** 10.3389/fmicb.2025.1664231

**Published:** 2025-11-05

**Authors:** Roberta Loddo, Matteo Incerti, Elda Favari, Valeria Manca, Marta Cogoni, Rebecca Piras, Luca Virdis, Vanessa Palmas, Elena Tamburini, Paolo La Colla, Giuseppina Sanna

**Affiliations:** ^1^Dipartimento di Scienze Biomediche, Sezione di Microbiologia e Virologia, Università degli Studi di Cagliari, Monserrato, Italy; ^2^Dipartimento di Scienze degli Alimenti e del Farmaco, Università degli Studi di Parma, Parma, Italy

**Keywords:** 1,2-benzisothiazol-3(2H)-one benzenesulfonamides, HIV, nucleocapsid proteins, NCp7, broad spectrum antiretroviral activity, mutant strains

## Abstract

A new class of 1,2-benzisothiazol-3(2H)-one benzenesulfonamides has been synthesized. In cell-based assays, the lead compound **6** inhibits the replication of HIV-1, HIV-2, and HIV-1 variants carrying clinically relevant mutations against non-nucleoside, nucleoside, and protease inhibitors. In enzyme assays, compound **6** does not inhibit HIV-1 reverse transcriptase and integrase. Genome sequencing of HIV-1 mutants selected for resistance to compound **6** reveals no mutations in the *pol* or *env* genes. Instead, two mutations are mapped in the gag region, which encodes nucleocapsid (NC) proteins involved in early and late key processes of retrovirus replication, suggesting that NC proteins are the target of the title compounds. Compound **6** shows concentration-dependent virucidal activity against cell-free HIV-1 and HIV-2. Benzisothiazol-3(2H)-one benzenesulfonamides are a new class of antiretroviral agents with an intriguing spectrum and mode of action.

## 1 Introduction

HIV infection leads to acquired immunodeficiency syndrome (AIDS) through progressive destruction of the immune system and degeneration of the central and peripheral nervous systems. One of the greatest achievements of modern medicine has been the development of antiretroviral therapy (ART) to prevent and treat HIV infection. However, the HIV pandemic is still increasing. Approximately 1.3 million people become infected with HIV each year, and about 630,000 die from HIV-related complications (World Health Organization, 2025). There is continued interest in the development of therapeutics against old and new targets in the HIV replication cycle, despite significant progress in the treatment of HIV/AIDS (Landovitz et al., 2023; Sever et al., 2024).

A large number of viral targets susceptible to selective inhibition or host-dependent factors have been identified (Puhl et al., 2019; Kinch and Patridge, 2014). These include the envelope CD4 binding protein gp120 and the fusion protein gp41; the key enzymes in HIV replication, reverse transcriptase (RT), integrase (IN), and protease (PR); regulatory proteins, such as TAT and C; and core proteins, such as the nucleocapsid NCp7 (De Clercq, 2002; Musah, 2004; Druillennec and Roques, 2000); however, the intrinsic high genetic variability of HIV combined with the error-prone nature of RT, which continuously generates mutants capable of overcoming inhibition by all known drugs, requires a combination of antiretroviral drugs with different modes of action.

The ability to target RT with two different classes of inhibitors, nucleoside (NRTIs) and non-nucleoside (NNRTIs), has made it possible to prove the efficacy of highly active antiretroviral therapy (HAART), initially based on triple combinations of drugs belonging to NRTIs, NNRTIs, and PR inhibitors (PRIs) (Flexner, 2007). Currently, NNRTIs, protease inhibitors, integrase inhibitors, and entry inhibitors are the five main classes of antiretroviral agents used in combination with ART (Melo et al., 2020; Shin et al., 2021).

For reasons such as virological failure (development of drug resistance and drug–drug interaction), adverse effects, convenience, or cost, regimens may need to be constantly updated (Gandhi et al., 2025; Battini and Bollini, 2019; Gill et al., 2019). Therefore, novel anti-HIV-1 agents are required to overcome these drawbacks.

**Figure 1:**
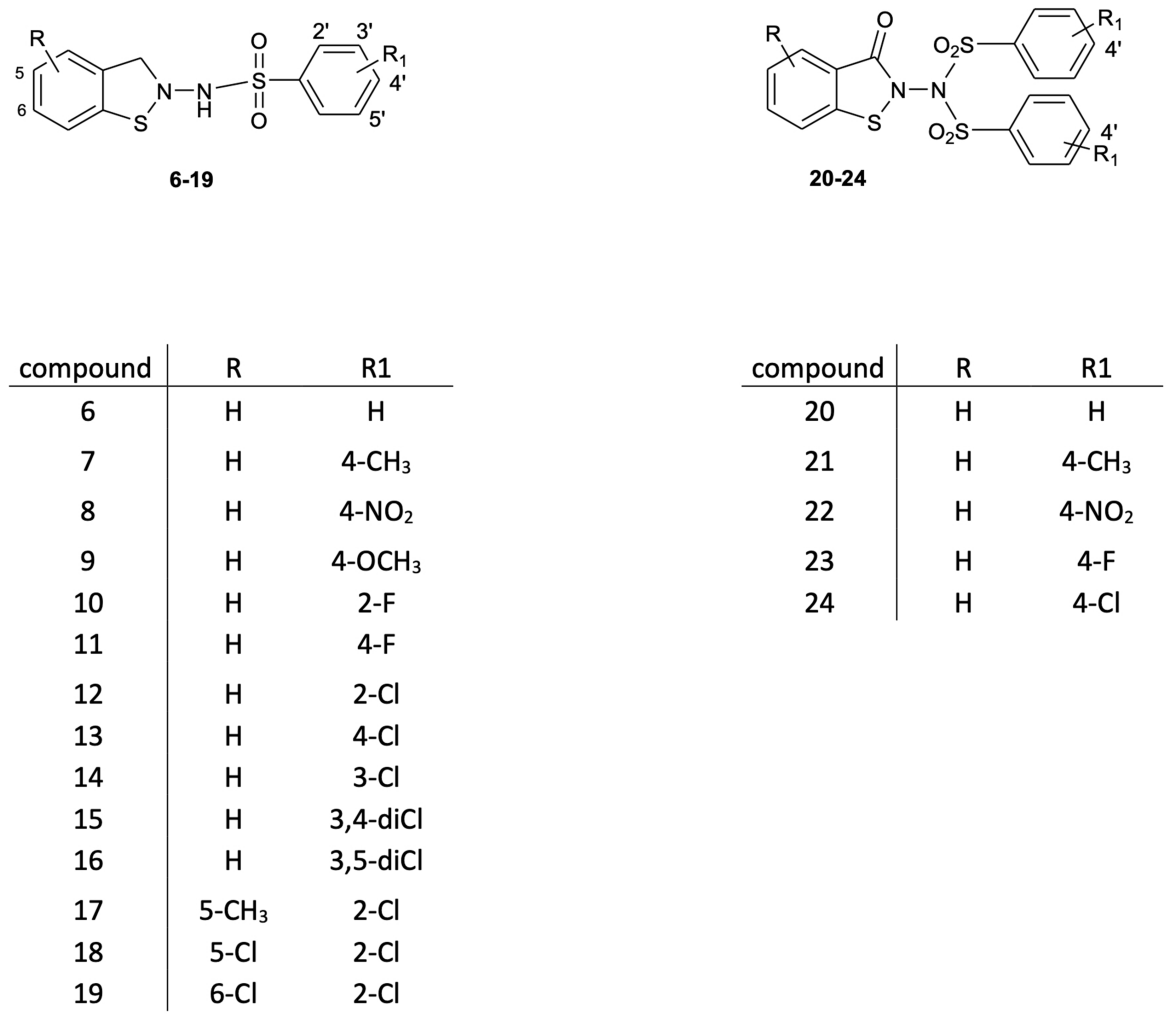
Chemical structure of 1,2-benzisothiazol-3(2H)-one benzensulfonamides **6–24**.

This study is an attempt to explore the potential of a new class of small molecule HIV inhibitors, 1,2-benzisothiazol-3(2H)-one benzenesulfonamides, which emerged from our research into biologically active benzisothiazole derivatives (Vicini et al., 2006; Vicini et al., 2007; Vicini et al., 2008; Vicini et al., 2009; Zani et al., 2009). The synthetic modulation of the basic scaffold, aimed at elucidating SAR analysis, led to structures **6–24** (Figure 1), which are endowed with antiretroviral activity *in vitro* at the micromolar level. Their potency is in the range reported by other researchers for benzisothiazolones and nucleocapsid (NC) inhibitors belonging to other classes (Goel et al., 2002; Sancineto et al., 2018). However, in addition to being capable of preventing the replication of HIV type-1 (HIV-1), type-2 (HIV-2), and HIV-1 strains resistant to the major drug classes currently used in clinics, these compounds show concentration-dependent inactivation of cell-free HIV-1 and HIV-2 infectivity in cell-based assays.

## 2 Materials and methods

### 2.1 Chemistry

Unless otherwise noted, reagents and solvents were purchased from commercial suppliers and used without purification. The progress of the reaction was monitored by thin-layer chromatography using F_254_ silica gel-precoated sheets, and spots were detected under a UV lamp at 254 nm. Flash chromatography was performed using Merck Silica Gel 60 (Si 60, 40–63 μm, 230–400 mesh ASTM; Merck KGaA, Darmstadt, Germany). Melting points were determined using a Gallenkamp melting point apparatus (Gallenkamp Labs, Cambridge, UK) and were not corrected. Infrared (IR) spectra were recorded as KBr pellets on a Jasco FT-IR 300E spectrophotometer (Jasco Ltd., Tokyo, Japan). Absorbance values are reported as *ν* (cm^−1^). ^1^H NMR spectra were recorded on a Bruker Avance 400 spectrometer (400 MHz; Bruker BioSpin GmbH, Rheinstetten, Germany). Chemical shifts (*δ* scale) are reported in parts per million (ppm). ^1^H NMR spectra were reported in the following order: multiplicity, approximate coupling constant (*J* value) in hertz (Hz), and number of protons. Signals were characterized as follows: s (singlet), d (doublet), t (triplet), and m (multiplet). Mass spectra were recorded on an API-150 EX system spectrometer (Applied Biosystems, MA, USA) with an electrospray ionization (ESI) interface. The final compounds were analysed for C, H, and N, and the percentages found were within ±0.4% of the theoretical values.

### 2.2 General experimental procedures

**SCHEME 1:**
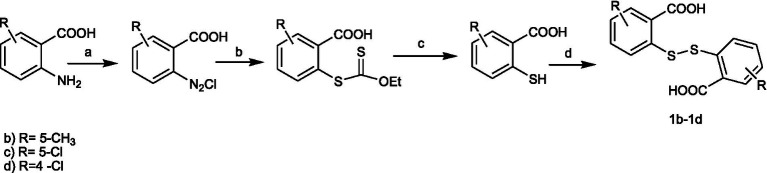
Synthesis of the key intermediates 1b–1d. Reagents and conditions: **(a)** NaNO2, HCl, 0°C; **(b)** C3H5KOS2; **(c)** NaOHaq 10%, reflux, 4h; HClconc; **(d)** I2. ethanol, 50 °C.

Bis(2-carboxyphenyl)disulphide (**1a**) was purchased from Sigma-Aldrich, while Bis(4-methyl-2-carboxyphenyl)disulphide (**1b**), Bis(4-chloro-2-carboxyphenyl)disulphide (**1c**), and Bis(5-chloro-2-carboxyphenyl)disulphide (**1d**) were prepared according to a modified procedure (Saha et al., 2017; Wu et al., 2023) outlined in Scheme 1. The suitably substituted 2-aminobenzoic acid was diazotized and then converted into an ester intermediate by a Sandmeyer reaction using potassium ethyloxantogenate. Next, upon alkaline hydrolysis and subsequent acidification, 2-mercaptoarylcarboxylic acid was obtained. Oxidation of the thiol group with iodine afforded key intermediates **1b-1d**.

**SCHEME 2:**
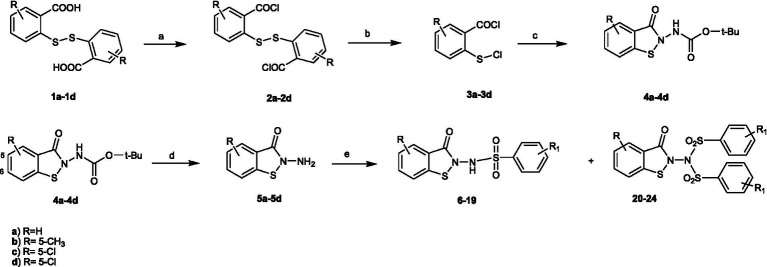
Synthesis of the target compounds **6-24**. Reagents and conditions: **(a)** SOCl_2_, reflux 3h; **(b)** Cl_2_, 3h, RT; **(c)** NH_2_-NH-Boc, CH_2_Cl_2_/pyridine,60 min. 10 °C; **(d)** CCl_3_COOH-H_2_O,150 min, rt; **(e)** R1- Phenyl-SO_2_Cl, pyridine –5 °C, 60 min.

From a modified procedure previously described (Yevich et al., 1986) and outlined in Scheme 2, the target compound **6** was synthesized, starting from Bis(2-carboxyphenyl)disulphide **1a** and treated with thionyl chloride to afford intermediate **2**, which was then converted into chlorocarbonylphenylsulfenylchloride **3** by treatment with dry chlorine. *Tert-butoxycarbonyl*hydrazine reacted with intermediate **3,** affording *tert*-Butyl-(3-oxo-1,2-benzisothiazol-2(*3H*)-yl)carbamate **4a**, which, after hydrolysis with trichloroacetic acid, yielded 2-amino-1,2-benzisothiazol-3(*2H*)-one **5a**, according to the method previously described by Vicini et al. (1997). Reaction with the appropriate benzenesulfonyl chloride, in pyridine, in a cooling bath, afforded a mixture of *N*-(3-oxo-1,2-benzisothiazol-2(*3H*)-yl)benzenesulfonamides **6** and of the respective *N*-(3-oxo-1,2-benzisothiazol-2(*3H*)-yl)-(phenylsulfonyl)benzenesulfonamide **20** (Figure 1).

Compounds **7–19** and **21–24** were synthesized following the previously described procedure for compounds **6** and **20,** starting from the suitably substituted Bis(2-carboxyphenyl)disulphide 1b–1d.

#### 2.2.1 Bis(4-methyl-2-carboxyphenyl)disulphide (**1b**)

Compound **1b** was synthesized following a previously described procedure (Saha et al., 2017; Wu et al., 2023), starting from 5-methyl-2-aminobenzoic acid.

#### 2.2.2 Bis(4-chloro-2-carboxyphenyl)disulphide (**1c**)

Compound **1c** was synthesized following a previously described procedure (Saha et al., 2017; Wu et al., 2023), starting from 5-chloro-2-aminobenzoic acid.

#### 2.2.3 Bis(5-chloro-2-carboxyphenyl)disulphide (**1d**)

Compound **1d** was synthesized following a previously described procedure (Saha et al., 2017; Wu et al., 2023), starting from 4-chloro-2-aminobenzoic acid.

#### 2.2.4 *tert*-Butyl *N-(*3-oxo-1,2-benzisothiazol-2(*3H*)-yl)carbamate ***4a***

Compound **4a** was synthesized following a previously described procedure (Vicini et al., 1997), starting from Bis(2-carboxyphenyl)disulphide (**1a**).

#### 2.2.5 *tert*-Butyl *N*- (5-methyl-3-oxo-1,2-benzisothiazol-2(*3H*)-yl)carbamate **4b**

Compound **4b** was synthesized following the procedure described for compound **4a,** starting from Bis(4-methyl-2-carboxyphenyl)disulphide (**1b**). Yield: 77%: White Crystals, mp 182–184 °C (ethyl acetate). ^1^H NMR (DMSO-d_6_): *δ* 10.02 (d, 1H, NH), 7.99 (d, 1H, *J* = 8.1, H-7), 7.71 (s, 1H, H-4), 7.54 (d, 1H, *J* = 8.1, H-6), 2.40 (s, 3H, CH_3_), 1.44 (s, 9H, CH_3_). IR (KBr): 3178, 2,979, 1745, 1,643. MS (ESI) calcd. For C_13_H_17_N_2_O_3_S [(M + 1)^+^]: 281.0960; found: 281.1. Anal. calcd. For C_13_H_16_N_2_O_3_S C,55.70; H,5.75; N,9.99; found C,55.85; H,5.46; N,9.67.

#### 2.2.6 *tert*-Butyl *N-*(5-chloro-3-oxo-1,2-benzisothiazol-2(*3H*)-yl)carbamate **4c**

Compound **4c** was synthesized following the procedure described for compound **4a,** starting from Bis(4-chloro-2-carboxyphenyl)disulphide (**1c**). Yield: 87%: White, pale yellowish solid, mp 164–165 °C (ethanol-water). ^1^H NMR (DMSO-d_6_): *δ* 10.11 (s, 1H, NH), 8.01 (d, 1H, *J* = 8.7, H-7), 7.89 (s, 1H, *J* = 1.8, H-4), 7.80 (dd, 1H, *J* = 1.8, *J* = 8.7, H-6), 1.44 (s, 9H, CH_3_). IR (KBr): 3186, 2,980, 1751, 1,643. MS (ESI) calcd. For C_12_H_14_ClN_2_O_3_S [(M + 1)^+^]: 301.0414; found: 301.7. Anal. calcd. For C_12_H_13_ClN_2_O_3_S C,47.92; H,4.36; N,9.31; found C,47.89; H,4.43; N,9.14.

#### 2.2.7 *tert*-Butyl *N*-(6-chloro-3-oxo-1,2-benzisothiazol-2(*3H*)-yl)carbamate **4d**

Compound **4d** was synthesized following the procedure described for compound **4a**, starting from Bis(5-chloro-2-carboxyphenyl)disulphide (**1d**). Yield: 60%, White solid, mp 156–157 °C (ethanol-water). ^1^H NMR (DMSO-d_6_): *δ* 10.08 (s, 1H, NH), 8.08 (s,1H, *J* = 2.1, H-7), 7.88 (d,1H, *J* = 8.4, H-4), 7.50 (d, 1H, *J* = 2.1, *J* = 8.7, H-5), 1.44 (s, 9H, CH_3_). IR (KBr): 3209, 2,978, 1755, 1,650. MS (ESI) calcd. For C_12_H_14_ClN_2_O_3_S [(M + 1)^+^]: 301.0414; found: 301.7. Anal. calcd. For C_12_H_13_ClN_2_O_3_S C,47.92; H,4.36; N,9.31; found C,48.02; H,4.47; N,9.08.

#### 2.2.8 2-Amino-1,2-benzisothiazol-3(*2H*)-one **5a**

Compound **5a** was synthesized following a previously described procedure (Vicini et al., 1997), starting from *tert*-Butyl *N*-(3-oxo-1,2-benzisothiazol-2(*3H*)-yl)carbamate **4a**.

#### 2.2.9 2-Amino-5-methyl-1,2-benzisothiazol-3(*2H*)-one **5b**

Compound **5b** was synthesized following a previously described procedure (Vicini et al., 1997), starting from *tert*-Butyl *N-*(5-methyl-3-oxo-1,2-benzisothiazol-2(*3H*)-yl)carbamate **4b**.

#### 2.2.10 2-Amino-5-chloro-1,2-benzisothiazol-3(*2H*)-one **5c**

Compound **5c** was synthesized following a previously described procedure (Vicini et al., 1997), starting from *tert*-Butyl N-(5-chloro-3-oxo-1,2-benzisothiazol-2(*3H*)-yl)carbamate **4c**. Yield: 47%, white solid, mp 140–141 °C (ethanol). ^1^H NMR (DMSO-d_6_): *δ* 7.93 (d, 1H, *J* = 8.4, H-7), 7.82 (s, 1H, *J* = 2.1, H-4), 7.72 (d, 1H, *J* = 2.1, *J* = 8.7, H-6), 5.65 (s, 2H, NH_2_). IR (KBr): 3305, 3,188, 1,676. MS (ESI) calcd. For C_7_H_6_ClN_2_OS [(M + 1)^+^]: 200.9889; found: 201.6. Anal. calcd. For C_7_H_5_ClN_2_OS C,41.90; H,2.51; N,13.96; found C,42.02; H,4.2.56; N,13.83.

#### 2.2.11 2-Amino-6-chloro-1,2-benzisothiazol-3(*2H*)-one **5d**

Compound **5d** was synthesized following a previously described procedure (Vicini et al., 1997), starting from *tert*-Butyl *N*-(6-chloro-3-oxo-1,2-benzisothiazol-2(*3H*)-yl)carbamate **4d**. Yield: 22%, pale yellow-pinkish crystals, mp 170–172 °C (ethanol). ^1^H NMR (DMSO-d_6_): *δ* 8.04 (s, 1H, *J* = 1.8, H-7), 7.82 (d, 1H, *J* = 1.8, H-4), 7.43 (dd, 1H, *J* = 1.8, *J* = 8.4, H-5), 5.07 (s, 2H, NH_2_). IR(KBr): 3296, 3,176, 1,653. MS (ESI) calcd. For C_7_H_6_ClN_2_OS [(M + 1)^+^]: 200.9889; found: 201.3. Anal. calcd. For C_7_H_5_ClN_2_OS C,41.90; H,2.51; N,13.96; found C,42.18; H,0.2.81; N,13.56.

#### 2.2.12 *N*-(3-oxo-1,2-benzisothiazol-2(*3H*)-yl)benzenesulfonamide 6 and *N*-(3-oxo-1,2-benzisothiazol-2(*3H*)-yl)-*N*-(phenylsulfonyl)benzenesulfonamide **20**

2-amino-1,2-benzisothiazol-3(*2H*)-one (**5a**) (10 mmol) was dissolved in anhydrous pyridine (8 mL) and cooled to 0 °C. Benzenesulfonyl chloride (11 mmol) was slowly added to the stirred solution under a nitrogen atmosphere. After the addition was complete, the reaction mixture was warmed to room temperature and stirred for 2 h. Crushed ice was added, and the mixture was acidified with concentrated hydrochloric acid until a precipitate was formed. The resulting suspension was filtered under vacuum, and the residue was washed with water. The obtained solid was purified by flash chromatography[SiO_2_, CH_2_Cl_2_: EtOH 95:5] to obtain, in order of elution, the first fraction, which was identified as compound **20,** and the second fraction, which was identified as compound **6**.

*N*-(3-Oxo-1,2-benzisothiazol-2(*3H*)-yl)-*N*-(phenylsulfonyl) benzenesulfonamide **20**. Yield: 40%. Pale yellow solid, mp 195–197 °C (ethanol). ^1^H NMR (DMSO-d_6_): *δ* 8.00–7.86 (m, 8H, H-4, H-7, H-2′, H-4′, H-6′, H-2″, H-4″, H-6″), 7.80 (td, 1H, *J* = 1.2, *J* = 8.1, H-5), 7.72 (t, 4H, *J* = 1.6, H-3′, H-5′, H-3″, H-5″), 7.47 (t, 1H, *J* = 1.6, H-6). ^13^C NMR (151 MHz, DMSO-d_6_) δ 164.10, 141.63, 137.12, 135.59, 134.15, 129.67, 128.76, 126.84, 126.13, 122.65, 119.62. IR (KBr): 3065, 1,688, 1,381, 1,173. MS (ESI) calcd. For C_19_H_15_N_2_O_5_S_3_ [(M + 1)^+^]: 447.0143; found: 447.1. Anal. calcd. For: C_19_H_14_N_2_O_5_S_3_ C,51.11; H,3.16; N,6.27; found C,51.27; H,3.22; N,5.88.

*N*-(3-Oxo-1,2-benzisothiazol-2(*3H*)-yl)benzenesulfonamide **6**. Yield: 30%. White solid, mp 162–164 °C (ethanol). ^1^H NMR (DMSO-d_6_): *δ* 11.34 (s, 1H, NH), 7.90–7.80 (m, 4H, H-4, H-7, H-2′, H-6′), 7.76–7.60 (m, 4H, H- H-5, H-6, H-3′, H-5′), 7.41 (t, 1H, *J* = 7.5, H-4′). ^13^C NMR (151 MHz, DMSO-d_6_) δ 163.17, 140.32, 138.85, 133.78, 133.00, 129.35, 127.65, 126.14, 126.72, 122.13, 121.24. IR (KBr): 3187, 3,062, 1,686, 1,348, 1,173. MS (ESI) calcd. For C_13_H_9_N_2_O_3_S_2_ [(M-1)^−^]: 305.0055; found: 305.5. Anal. calcd. For C_13_H_10_N_2_O_3_S_2_ C,50.97; H,3.29; N,9.14; found C,51.16; H,3.37; N,9.14.

#### 2.2.13 4-Methyl-*N*-(3-oxo-1,2-benzisothiazol-2(*3H*)-yl)benzenesulfonamide 7 and 4-methyl-*N*-[(4-methylphenyl)sulfonyl]-*N*-(3-oxo-1,2-benzisothiazol-2(*3H*)-yl)benzenesulfonamide **21**

Compounds **7** and **21** were synthesized following the same procedure previously described for compounds **6** and **20**.

4-Methyl-*N*-[(4-methylphenyl)sulfonyl]-*N*-(3-oxo-1,2-benzisothiazol-2(*3H*)-yl)benzenesulfonamide **21**. Yield: 40%. White solid, mp 203–204 °C (ethanol). ^1^H NMR (DMSO-d_6_): *δ* 7.98 (d, 1H, *J* = 8.1, H-4), 7.92 (d, 1H, *J* = 7.8, H-7), 7.85–7.76 (m, 5H, H-5, H-2′, H-6′, H-2″, H-6″), 7.53–7.41 (m, 5H, H-6, H-3′, H-5′, H-3″, H-5″), 2.46 (s, 6H, CH_3_). ^13^C NMR (151 MHz, DMSO-d_6_) δ 164.12, 146.50, 141.61, 134.33, 134.07, 130.06, 128.82, 126.83, 126.07, 122.61, 119.70, 21.26. IR (KBr), 3,056, 2,920, 2,854, 1705, 1,361, 1,170. MS (ESI) calcd. For C_21_H_19_N_2_O_5_S_3_ [(M + 1)^+^]: 475.0456; found: 475.0. Anal. calcd. For C_21_H_18_N_2_O_5_S_3_ C,53.15; H,3.82; N,5.90; found C,53.53; H,3.90; N,5.90.

4-Methyl-*N*-(3-oxo-1,2-benzisothiazol-2(*3H*)-yl)benzenesulfonamide **7**. Yield: 28%. Pale yellow crystals, mp 168–170 °C (ethanol). ^1^H NMR (DMSO-d_6_): *δ* 11.21 (s, 1H, NH), 7.87 (d, 1H, *J* = 8.1, H-4), 7.81 (d, 1H, *J* = 8.1, H-7), 7.77–7.67 (m, 3H, H-5, H-2′, H-6′), 7.44–7.38 (m, 3H, H-6, H-3′, H-5′), 2.41 (s, 3H, CH_3_). ^13^C NMR (151 MHz, DMSO-d_6_) δ 163.20, 144.22, 140.31, 135.99, 132.96, 129.81, 127.72, 126.14, 125.69, 122.13, 121.29, 21.12. IR (KBr): 3057, 2,850, 1,656, 1,340, 1,158. MS (ESI) calcd. For C_14_H_11_N_2_O_3_S_2_ [(M-1)^−^]: 319.0211; found: 319.2. Anal. calcd. For C_14_H_12_N_2_O_3_S_2_ C,52.49; H,3.78; N,8.74; found C,52.43; H,3.72; N,8.48.

#### 2.2.14 4-Nitro-*N*-(3-oxo-1,2-benzisothiazol-2(*3H*)-yl)benzenesulfonamide 8 and 4-Nitro-*N*-[(4-nitrophenyl)sulfonyl]-*N*-(3-oxo-1,2-benzisothiazol-2(*3H*)-yl)benzenesulfonamide **22**

Compounds **8** and **22** were synthesized following the same procedure described for compounds **6** and **20**.

4-Nitro-*N*-[(4-nitrophenyl)sulfonyl]-*N*-(3-oxo-1,2-benzisothiazol-2(*3H*)-yl)benzenesulfonamide **22**. Yield: 30%. Yellow solid, mp 194–195 °C (acetic acid). ^1^H NMR (DMSO-d_6_): *δ* 8.53 (d, 4H, *J* = 8.7, H-3′, H-5′, H-3″, H-5″), 8.24 (d, 4H, *J* = 9.3, H-2′, H-6′, H-2″, H-6″), 8.02 (d, 1H, *J* = 8.1, H-4), 7.92 (d, 1H, *J* = 7.5, H-7), 7.84 (t, 1H, *J* = 8.1, H-5), 7.50 (t, 1H, *J* = 7.5, H-6). ^13^C NMR (151 MHz, DMSO-d_6_) *δ* 164.29, 154.33, 151.45, 147.26, 141.89, 141.77, 134.52, 130.72, 127.07, 126.91, 126.58, 125.07, 123.32, 122.87, 119.38. IR (KBr): 3112, 1704, 1,535, 1,376, 1,348, 1,182. MS (ESI) calcd. For C_19_H_13_N_4_O_9_S_3_ [(M + 1)^+^]: 536.9845; found: 536.1. Anal. calcd. For C_19_H_12_N_4_O_9_S C,42.53; H,2.25; N,10.44; found C,42.44; H,2.26; 10.45.

#### 2.2.15 4-Nitro-*N*-(3-oxo-1,2-benzisothiazol-2(*3H*)-yl)benzenesulfonamide **8**

Yield: 47%. Yellow solid, mp 176–178 °C (ethanol). ^1^H NMR (DMSO-d_6_): *δ* 11.34 (s, 1H, NH), 8.43 (d, 2H, *J* = 9.0, H-3′, H-5′), 8.12 (d, 2H, *J* = 8.4, H-2′, H-6′), 7.91 (d, 1H, *J* = 8.1, H-4), 7.79 (d, 1H, *J* = 8.1, H-7), 7.72 (t, 1H, *J* = 8.1, H-5), 7.42 (t, 1H, *J* = 7.0, H-6). ^13^C NMR (151 MHz, DMSO-d_6_) δ 163.20, 150.30, 144.66, 140.47, 133.18, 129.42, 126.22, 125.84, 124.57, 122.21, 121.06. IR (KBr): 3033, 2,846, 1,655, 1,354, 1,169. MS (ESI) calcd. For C_13_Hc._8_N_3_O_5_S_2_ [(M-1)^−^]: 349.9905; found: 350.8. Anal. calcd. For C_13_H_9_N_3_O_5_S_2_ C,44.44; H,2.58; N,11.96; found C,44.57; H,2.74; 11.61.

#### 2.2.16 4-Methoxy-*N*-(3-oxo-1,2-benzisothiazol-2(*3H*)-yl)benzenesulfonamide **9**

Compound **9** was synthesized following the same procedure described for compound **6**.

Yield: 27%. Pale yellow solid, mp 167 °C (ethanol-water). ^1^H NMR (DMSO-d_6_): *δ* 11.13 (s, 1H, NH), 7.88 (d, 1H, *J* = 8.1, H-4), 7.84–7.76 (m, 3H, H-7, H-2′, H-6′), 7.70 (t, 1H, *J* = 7.8, H-5), 7.41 (t, 1H, *J* = 7.8, H-6), 7.15 (d, 2H, *J* = 9.0, H-3′, H-5′), 3.85 (s, 3H, CH_3_). ^13^C NMR (151 MHz, DMSO-d_6_) δ 163.23, 163.16, 140.31, 132.93, 130.06, 126.13, 125.67, 122.11, 121.31, 114.61, 114.55, 55.74. IR (KBr): 3060, 2,841, 1,670, 1,346, 1,155. MS (ESI) calcd. For C_14_H_11_N_2_O_4_S_2_ [(M-1)^−^]: 335.0160; found: 335.1. Anal. calcd. For C_14_H_12_N_2_O_4_S C,49.99; H,3.60; N,8.33; found C,50.34; H,3.70; N 8.31.

Anal: C_14_H_12_N_2_O_4_S_2_ (336.39).

#### 2.2.17 2-Fluoro-*N*-(3-oxo-1,2-benzisothiazol-2(*3H*)-yl)benzenesulfonamide **10**

Compound **10** was synthesized following the same procedure described for Compound **6**. Yield: 22%. Ivory solid, mp 159–161 °C (ethanol). ^1^H NMR (DMSO-d_6_): *δ* 11.67 (s, 1H, NH), 7.88 (d, 1H, *J* = 7.5, H-4), 7.82–7.68 (m, 4H, H-7, H-3′, H-4′, H-6′), 7.50 (t, 1H, H-5), 7.43–7.33 (m, 2H, H-6, H-5′). IR (KBr): 3060, 2,829, 1,664, 1,356, 1,176. MS (ESI) calcd. For C_13_H_8_FN_2_O_3_S_2_ [(M-1)^−^]: 322.9960; found: 323.8. Anal. calcd. For C_13_H_9_FN_2_O_3_S_2_C,48.14; H,2.80; N,8.64; found C,48.01; H,3.08; N 8.36.

#### 2.2.18 4-Fluoro-*N*-(3-oxo-1,2-benzisothiazol-2(*3H*)-yl)benzenesulfonamide 11 and 4-Fluoro-*N*-[(4-fluorolphenyl)sulfonyl]-*N*-(3-oxo-1,2-benzisothiazol-2(*3H*)-yl)benzenesulfonamide **23**

Compounds **11** and **23** were synthesized following the same procedure described for compounds **6** and **20**.

#### 2.2.19 4-Fluoro-*N*-[(4-fluorolphenyl)sulfonyl]-*N*-(3-oxo-1,2-benzisothiazol-2(*3H*)-yl)benzenesulfonamide **23**

Yield: 33%. Ivory solid, mp 195–196 °C (ethanol). ^1^H NMR (DMSO-d_6_): *δ* 8.10–8.01 (m, 4H, H-2′, H-6′, H-2″, H-6″), 7.98 (d, 1H, *J* = 8.1, H-4), 7.91 (d, 1H, *J* = 8.4, H-7), 7.81 (td, 1H, *J* = 8.1, *J* = 7.2, H-5), 7.64–7.56 (m, 4H, H-3′, H-5′, H-3″, H-5″), 7.48 (td, 1H, *J* = 0.9, *J* = 7.8, H-6). ^13^C NMR (151 MHz, DMSO-d_6_) *δ* 166.84, 165.15, 164.20, 141.66, 134.23, 133.27, 132.31, 127.89, 126.19, 122.71, 119.58, 117.15. IR (KBr): 3187, 3,062, 1,686, 1,348, 1,173. MS (ESI) calcd. For C_19_H_13_F_2_N_2_O_5_S_3_ [(M + 1)^+^]: 482.9955; found: 483.6. Anal. calcd. For C_19_H_12_F_2_N_2_O_5_S_3_ C,47.30; H,2.51; N, 5.81; found C,47.65; H,3.2.52; N 5.65.

#### 2.2.20 4-Fluoro-*N*-(3-oxo-1,2-benzisothiazol-2(*3H*)-yl)benzenesulfonamide **11**

Yield: 50%. Ivory solid, mp 150–151 °C (methanol–water). ^1^H NMR (DMSO-d_6_): *δ* 11.39 (s, 1H, NH), 7.96–7.88 (m, 3H, H-4, H-2′, H-6′), 7.80 (d, 1H, *J* = 8.4, H-7), 7,71 (td, 1H, *J* = 1.5, *J* = 7.2, H-5), 7.51–7.38 (m, 3H, H-6, H-3′, H-5′). ^13^C NMR (151 MHz, DMSO-d_6_) δ 165.49, 163.15, 140.37, 135.16, 135.14, 133.04, 130.95, 125.95, 122.16, 121.21, 116.59. IR (KBr): 3048, 2,848, 1,656, 1,355, 1,161. MS (ESI) calcd. For C_13_H_8_FN_2_O_3_S_2_ [(M-1)^−^]: 322.9960; found: 323.2. Anal. calcd. For C_13_H_9_FN_2_O_3_S_2_C,48.14; H,2.80; N,8.64; found C,48.56; H,2.81; N 8.22.

#### 2.2.21 2-Chloro-*N*-(3-oxo-1,2-benzisothiazol-2(*3H*)-yl)benzenesulfonamide **12**

Compound **12** was synthesized following the same procedure described for compound **6**. Yield: 33%. Ivory solid, mp 170–172 °C (ethanol). ^1^H NMR (DMSO-d_6_): *δ* 11.57 (s, 1H, NH), 7.98 (d, 1H, *J* = 8.7, H-4), 7.87 (d, 1H, *J* = 7.8, H-6′), 7.82 (d, 1H, *J* = 8.4, H-7), 7.77–7.67 (m, 3H, H-5, H-3′, H-5′), 7.51 (t, 1H, H-4′), 7.40 (t, 1H, *J* = 7.5, H-6). ^13^C NMR (151 MHz, DMSO-d_6_) *δ* 163.32, 140.41, 136.56, 135.16, 133.05, 132.01, 131.83, 131.40, 127.70, 126.17, 125.74, 122.14, 121.15. IR (KBr): 3018, 2,850, 1,672, 1,344, 1,170. MS (ESI) calcd. For C_13_H_8_ClN_2_O_3_S_2_ [(M-1)^−^]: 338.9665; found: 339.2. Anal. calcd. For C_13_H_9_FN_2_O_3_S_2_C,45.81; H,2.66; N,8.22; found C,45.41; H,2.82; N 8.10.

#### 2.2.22 4-Chloro-*N*-(3-oxo-1,2-benzisothiazol-2(*3H*)-yl)benzenesulfonamide 13 and 4-chloro-*N*-[(4-chlorophenyl)sulfonyl]-*N*-(3-oxo-1,2-benzisothiazol-2(*3H*)-yl)benzenesulfonamide **24**

Compounds **13** and **24** were synthesized following the same procedure described for compounds **6** and **20**.

#### 2.2.23 4-Chloro-*N*-[(4-chlorophenyl)sulfonyl]-*N*-(3-oxo-1,2-benzisothiazol-2(*3H*)-yl)benzenesulfonamide **24**

Yield: 80%. Pale yellow solid, mp 222–224 °C dec.(ethanol). ^1^H NMR (DMSO-d_6_): *δ* 8.04–7.98 (m, 5H, H-4, H-2′, H-6′, H-2″, H-6″), 7.92 (d, 1H, *J* = 8.1, H-7), 7.86–7.78 (m, 5H, H-5 H-3′, H-5′, H-3″, H-5″), 7.49 (t, 1H, *J* = 7.8, H-6). IR (KBr): 3057, 2,850, 1,656, 1,340, 1,158. MS (ESI) calcd. For C_19_H_13_Cl_2_N_2_O_5_S_3_ [(M + 1)^+^]: 514.9364; found: 516.5. Anal. calcd. For C_19_H_12_Cl_2_N_2_O_5_S_3_C,44.36; H,2.35; N,5.45; found C,44.47; H,2.38; N 5.44.

#### 2.2.24 4-Chloro-*N*-(3-oxo-1,2-benzisothiazol-2(*3H*)-yl)benzenesulfonamide **13**

Yield: 28%. Pale yellow solid, mp 158–159 °C (ethanol). ^1^H NMR (DMSO-d_6_): *δ* 11.58 (s, 1H, NH), 7.91–7.81 (m, 4H, H-4, H-7, H-2′, H-6′), 7.74–7.68 (m, 3H, H-5, H-3′, H-5′), 7.42 (t, 1H, *J* = 7.1, 6). ^13^C NMR (151 MHz, DMSO-d_6_) δ 163.17, 140.38, 138.73, 137.81, 133.06, 129.66, 129.52, 126.18, 125.76, 122.18, 121.19. IR (KBr): 3058, 2,852, 1,652, 1,342, 1,161. MS (ESI) calcd. For C_13_H_8_ClN_2_O_3_S_2_ [(M-1)^−^]: 338.9665; found: 339.6. Anal. calcd. For C_13_H_9_FN_2_O_3_S_2_C,45.81; H,2.66; N,8.22; found C,45.63; H,2.98; N 8.04.

#### 2.2.25 3-Chloro-*N*-(3-oxo-1,2-benzisothiazol-2(*3H*)-yl)benzenesulfonamide **14**

Compound **14** was synthesized following the same procedure described for compound **6**. Yield: 23%. Tan solid, mp 149–150 °C (Ethanol). ^1^H NMR (DMSO-d_6_): *δ* 11.48 (s,1H, NH), 7.91–7.81 (m, 4H, H-4, H-7, H-2′, H-6′), 7.74–7.73 (m, 3H, H-5, H-3’ H-5′), 7.42 (t, 1H, *J* = 7.6, H-6). ^13^C NMR (151 MHz, DMSO-d_6_) *δ* 163.18, 140.84, 140.39, 133.86, 133.75, 133.09, 131.38, 127.11, 126.43, 126.18, 125.79, 122.20, 121.16. IR (KBr): 3074, 2,870, 1,664, 1,365, 1,176. MS (ESI) calcd. For C_13_H_8_ClN_2_O_3_S_2_ [(M-1)^−^]: 338.9665; found: 339.6. Anal. calcd. For C_13_H_9_FN_2_O_3_S_2_C,45.81; H,2.66; N,8.22; found C,45.88; H,2.61; N 8.07.

#### 2.2.26 3,4-Dichloro-*N*-(3-oxo-1,2-benzisothiazol-2(*3H*)-yl)benzenesulfonamide **15**

Compound **15** was synthesized following the same procedure described for compound **6**. Yield: 35%. Ivory solid, mp 183–184 °C (ethanol) ^1^H NMR (DMSO-d_6_): *δ* 11.69 (s,1H, NH), 8.05 (s, 1H, *J* = 7.6, H-2′), 7.92–7.89 (m, 2H, H-4, H-6′), 7.84–7.79 (m, 2H, H-7, H-5′), 7.72 (td, 1H, *J* = 1.2, *J* = 8.2, H-5), 7.42 (t, 1H, *J* = 7.3, H-6). HS17 (15). ^13^C NMR (151 MHz, DMSO-d_6_) *δ* 163.20, 140.45, 139.36, 136.90, 133.14, 132.19, 131.72, 129.32, 127.84, 126.21, 125.82, 122.23, 121.12. IR (KBr): 3031, 2,852, 1,657, 1,327, 1,169. MS (ESI) calcd. For C_13_H_7_Cl_2_N_2_O_3_S_2_ [(M-1)^−^]: 372.9275; found: 373.4. Anal. calcd. For C_13_H_8_Cl_2_N_2_O_3_S_2_ C,41.61; H,2.15; N,7.47; found C,41.96; H,2.20; N 7.42.

#### 2.2.27 3,5-Dichloro-*N*-(3-oxo-1,2-benzisothiazol-2(*3H*)-yl)benzenesulfonamide **16**

Compound **16** was synthesized following the same procedure above for compound **6**.

Yield: 28%. Pale yellow solid, mp 178–180 °C (ethanol-water). ^1^H NMR (DMSO-d_6_): *δ* 11.50 (s, 1H, NH), 8.06 (t, 1H, *J* = 1.9, H-4′), 7.92 (d, 1H, *J* = 8.4, H-4), 7.84–7.82 (m, 3H, H-7, H-2′, H-6′), 7.73 (td, 1H, *J* = 1.5, *J* = 8.7, H-5), 7.42 (t, 1H, *J* = 7.2, H-6). IR (KBr): 3216, 3,085, 1,672, 1,362, 1,178. MS (ESI) calcd. For C_13_H_7_Cl_2_N_2_O_3_S_2_ [(M-1)^−^]: 372.9275; found: 373.2. Anal. calcd. For C_13_H_8_Cl_2_N_2_O_3_S_2_ C,41.61; H,2.15; N,7.47; found C,41.23; H,2.53; N 7.04.

#### 2.2.28 2-Chloro-*N*-(5-methyl-3-oxo-1,2-benzisothiazol-2(*3H*)-yl)benzenesulfonamide **17**

Compound **17** was synthesized following the same procedure described for compound **6**. Yield: 35%. Ivory solid, mp 174–176 °C (ethanol). ^1^H NMR (DMSO-d_6_): *δ* 11.51 (s, 1H, NH), 7.94 (d, 1H, *J* = 7.5, H-6′), 7.76–7.68 (m, 3H, H-6, H-7, H-3′), 7.62 (s,1H, 4), 7.55–7.47 (m, 2H, H-3′, H-4′), 2.37 (s, 3H, CH_3_). ^13^C NMR (151 MHz, DMSO-d_6_) *δ* 163.29, 137.45, 136.58, 135.46, 135.12, 134.38, 131.99, 131.84, 131.38, 127.67, 125.84, 121.89, 121.17. IR (KBr): 3022, 2,781, 1,668, 1,342, 1,169. MS (ESI) calcd. For C_14_H_10_ClN_2_O_3_S_2_ [(M-1)^−^]: 352.9821; found: 352.1. Anal. calcd. For C_14_H_11_ClN_2_O_3_S_2_ C,47.39; H,3.12; N,7.89; found C,47.46; H,3.25; N,7.80.

#### 2.2.29 2-Chloro-*N*-(5-chloro-3-oxo-1,2-benzisothiazol-2(*3H*)-yl)benzenesulfonamide **18**

Compound **18** was synthesized following the same procedure described for compound **6**. Yield: 23%. Tan solid, mp 174–175 °C (Ethanol). ^1^H NMR (DMSO-d_6_): *δ* 11.57 (s, 1H, NH), 7.99 (d, 1H, *J* = 8.1, H-6′), 7.93 (d, 1H, *J* = 8.7, H-7), 7.80 (d, 1H, *J* = 2.2, H-4), 7.76–7.69 (m, 3H, H-6, H-5′, H-3′), 7.50 (td, 1H, *J* = 1.6, *J* = 8.1, H-4′). IR (KBr): 3035, 2,852, 1,662, 1,350, 1,167. MS (ESI) calcd. For C_13_H_7_Cl_2_N_2_O_3_S_2_ [(M-1)^−^]: 372.9275; found: 373.7. Anal. calcd. For C_13_H_8_Cl_2_N_2_O_3_S_2_ C,41.61; H,2.15; N,7.47; found C,41.90; H,2.47; N 7.11.

#### 2.2.30 2-Chloro-*N*-(6-chloro-3-oxo-1,2-benzisothiazol-2(*3H*)-yl)benzenesulfonamide **19**

Compound **19** was synthesized following the same procedure described for compound **6**. Yield: 23%. Pale yellow solid, mp 153–155 °C (Ethanol). ^1^H NMR (DMSO-d_6_): *δ* 11.61 (s, 1H, NH), 8.00–7.96 (m, 2H, H-6′, H-7), 7.79 (d, 1H, *J* = 8.1, H-4), 7.75–7.67 (m, 2H, H-5, H-5′), 7.53–7.42 (m, 2H, H-3′, H-4′). ^13^C NMR (151 MHz, DMSO-d_6_) δ 162.49, 141.93, 137.96, 136.57, 135.16, 132.02, 131.80, 131.41, 127.72, 126.26, 121.79, 120.23. IR (KBr): 3091, 2,850, 1,664, 1,358, 1,176. MS (ESI) calcd. For C_13_H_7_Cl_2_N_2_O_3_S_2_ [(M-1)^−^]: 372.9275; found: 373.1. Anal. calcd. For C_13_H_8_Cl_2_N_2_O_3_S_2_ C,41.61; H,2.15; N,7.47; found C,41.90; H,2.50; N 7.05.

### 2.3 Biology

#### 2.3.1 Compounds

Compounds were solubilized in DMSO at 100 mM and then diluted in a culture medium. The final concentration of DMSO in samples and controls employed in biological assays was less than 0.02%.

#### 2.3.2 Cells and viruses

MT-4, C8166, H9/IIIB, and CEM cells, as well as laboratory-adapted HIV-1 strains, were obtained from the NIH AIDS Research &Reference Reagent Program, USA. Cell cultures were grown in RPMI 1640 medium, supplemented with 10% foetal calf serum (FCS), 100 IU/mL penicillin G, and 100 μg/mL streptomycin, and incubated at 37 °C in a 5% CO_2_ atmosphere. Cell cultures were checked periodically for the absence of mycoplasma contamination using the MycoTect Kit (Gibco, Thermo Fisher Scientific, MA, USA).

HIV-1_IIIB_ and HIV-2 CBL-20 strains were obtained from the supernatants of persistently infected H9/IIIB and CEM cells, respectively. HIV-1 and HIV-2 stock solutions had titres of 4.5 × 10^7^ and 1.4 × 10^6^ 50% cell culture infectious doses (CCID_50_)/ml, respectively. The Y181C mutant (NIH N119) was derived from an AZT-sensitive clinical isolate passaged initially in CEM and then in MT-4 cells in the presence of Nevirapine (up to 10 μM). The K103N + Y181C mutant (NIH A17) was derived from an IIIB strain passaged in H9 cells in the presence of BI-RG 587 (up to 1 μM). The K103R + V179D + P225H mutant (EFVR) was derived from an IIIB strain passaged in MT-4 cells in the presence of Efavirenz (up to 2 μM). N119, A17 and EFV^R^ stock solutions had titres of 1.2 × 10^8^, 2.1 × 10^7^, and 4.0 × 10^7^ CCID_50_/ml, respectively. Mutants carrying NRTI mutations, such as the AZT^R^ strain (67N, 70R, 215F, 219Q) and the MDR strain (74V, 41L, 106A, 215Y) or PRI mutations, such as SAQ^R^ (L10F, G48V, L90M), were also tested.

#### 2.3.3 Biological evaluation of antiviral activities

##### 2.3.3.1 Anti-HIV assays

The activity of the test compounds against the replication of HIV-1 wt and mutant strains (N119, A17, EFV^R^, AZT^R^, MDR, SAQ^R^) and HIV-2 wt in acutely infected cells was based on the inhibition of virus-induced cytopathogenicity in MT-4 and C8166 cells, respectively. Briefly, 50 μL of culture medium containing 1 × 10^4^ cells was added to each well of flat-bottom microtitre trays containing 50 μL of culture medium with or without different serial concentrations of test compounds. Then, 20 μL of an HIV suspension (containing the appropriate amount of CCID_50_ required to cause complete cytopathogenicity on day 4, final multiplicity of infection [m.o.i.] 0.01) was added. After incubation at 37 °C, cell viability was determined using the 3-(4,5-dimethylthiazol-1-yl)-2,5-diphenyltetrazolium bromide (MTT) method (Pauwels et al., 1988). The cytotoxicity of the test compounds was evaluated in parallel with their antiviral activity through the viability of mock-infected, treated cells, as monitored by the MTT method and described elsewhere (Morellet et al., 1994).

#### 2.3.4 Mode of action studies

##### 2.3.4.1 Reverse transcriptase assay

Assays using recombinant HIV-1 reverse transcriptase were performed as previously described (Costi et al., 2004; Tramontano and Cheng, 1992).

##### 2.3.4.2 Integrase assays

Expression and purification of HIV-1 recombinant integrase (rIN) and rIN assays were performed as previously reported (Costi et al., 2004).

#### 2.3.5 p24 determination

Quantitation of the p24 Gag protein present in supernatants of HIV-1-infected MT-4 cells was assessed using the Alliance HIV-1 P24 ANTIGEN ELISA Kit (Perkin Elmer), according to the manufacturer’s protocol, on culture supernatant using antigen-capture enzyme-linked immunosorbent assay test (ELISA), Perkin Elmer.

##### 2.3.5.1 H9/C8166 cocultures

Chronically infected H9 cells were washed and cocultured with uninfected C8166 cells (ratio 1:500) in the absence or presence of test inhibitors. Following 36 h of incubation at 37 °C, cocultures were monitored by optical microscopy, syncytia were counted, and those found in drug-treated cocultures were reported as a percentage of those counted in untreated cocultures (Wang et al., 2004).

##### 2.3.5.2 Time of addition assay

2 × 10^4^ MT-4 cells were infected for 1 h at 20 °C with HIV-1 at a high multiplicity of infection (m.o.i. = 5), corresponding to 5 cell culture infectious dose 50 per cell (CCID_50_/cell). Test compounds were added in duplicate at the beginning of the 1 h infection period (and then removed with the inoculum by extensive washing) or immediately after the end of infection, 1, 2, 4, 6, 8, 12, or 24 h later. The amounts of p24 and infectious virus present in the supernatants were measured at 42 h post-infection (p.i.).

##### 2.3.5.3 Selection of drug-resistant mutants

Drug-resistant variants were selected by serial passages of HIV-1_IIIB_ in the presence of progressively doubling drug concentrations, starting from a cell culture infected with an m.o.i. of 0.01 and treated with a drug concentration equal to the EC_50_. Usually, the amount of virus obtained after each passage was sufficient to determine the infection of the next cell culture, which, after infection and washing, was incubated with double the amount of the selected drug. In the case of compound **6**, the drug-resistant virus population was selected up to a drug concentration 8-fold greater than the EC_50_. Resistant virus preparations were subjected to RNA extraction, RT-PCR, and genome sequencing to identify the mutational patterns responsible for resistance.

##### 2.3.5.4 Molecular analysis of resistant viruses

Viral RNAs from wt and drug-resistant mutants were obtained using the QIAamp viral RNA minikit (Qiagen), starting from 140 μL of cell-free viral suspensions containing about 5 × 10^4^ PFU/ml, to determine the nucleotide sequence of the *pol*, *gag,* and *env* regions of the HIV-1 genome. Reverse transcriptions were carried out using the Superscript II enzyme (Invitrogen), and cDNAs were amplified by PCR using Platinum Pfx polymerase (Invitrogen) following the manufacturer’s protocol. Details of the RT-PCR conditions are provided in the Supplementary Data. PCR fragments were purified using the QIAquick PCR Purification kit (Qiagen) and analyzed using the cycle-sequencing method (C. I. B. I. A. C. I. service of the University of Florence). Both DNA strands were sequenced using specific primers. The comparative analysis of received chromatograms allowed us to obtain the nucleotide sequences of the *gag, pol,* and *env* regions for wild-type HIV_IIIB_ and HIV-1^**6**^ resistant.

##### 2.3.5.5 Virucidal activity assays

Cell-free, high-titre HIV-1 and HIV-2 stock solutions were exposed to the test compounds for 2 h at 0 °C or 37 °C. At the end of incubation, residual infectivity was determined using the Reed–Muench end-point titration method, as described below.

##### 2.3.5.6 HIV titration

Titration was performed in C8166 cells using the standard limiting dilution method (dilution 1:2, four replica wells/dilution) in 96-well plates. The infectious virus titre was determined by light microscope scoring of syncytia (multinucleated giant cells) after 4 days of incubation (Tondelli et al., 1996). Virus titres were expressed as 50% cell culture infectious dose per ml using the Reed and Muench (1938) method.

## 3 Results

### 3.1 Antiviral activities of benzenesulfonamide derivatives against HIV-1, HIV-2, and mutant strains

The cytotoxicity (CC_50_), potency (EC_50_), and spectrum of antiretroviral activity of 1,2- benzisothiazol-3(2H)-one benzenesulfonamides (derivatives 6–24) are reported in Table 1. The HIV-1 strains tested included wild-type _IIIB_ and mutants resistant to NNRTIs, NRTIs, and PRIs, as detailed in the Materials and Methods. Activity against HIV-2 (CBL-20 strain) in C8166 cells was also reported. In this study, benzenesulfonamides were evaluated in comparison with the selected reference drugs: efavirenz (EFV), azidothymidine (AZT), and saquinavir (SAQ). Most of the title derivatives were effective in the micromolar concentration range against HIV-1 wild-type and variants carrying clinically relevant mutations that confer resistance to the three representative classes of antiretrovirals. Furthermore, in contrast to EFV, compounds 6, 7, 17, and 19 inhibited HIV-2 as well as HIV-1 (Table 1), demonstrating their broad-spectrum antiretroviral activity and Selectivity Index (SI) of around 10 against all tested strains.

**Table 1:** Cytotoxicity and antiretroviral activity of compounds **6–24** and reference drugs.

Compounds	MT-4	^c^HIV-1	^d^N119	^d^A17	^d^EFV^R^	^e^AZT^R^	^e^MDR	^f^SAQ^R^	^g^HIV-2
	^a^CC_50_ (μM)	^b^EC_50_ (μM)							
**6**	86 ± 4	8 ± 3(10)	7 ± 1.5(12)	8 ± 1 (10)	7 ± 2 (12)	6 ± 0.7 (14)	4 ± 1.5 (21)	8 ± 0.7 (10)	7 ± 0.7 (12)
**7**	35 ± 1	10 ± 3 (3.5)	5 ± 0.1 (7)	8 ± 1 (4)	8 ± 3 (4)	8 ± 1 (4)	6 ± 0.7 (5)	9 ± 2 (3)	4 ± 0.7 (8)
**8**	>100	15 ± 4(>7)	7 ± 0.5(>14)	11 ± 2(>9)	9 ± 2(>11)	7 ± 0.7 (14)	6 ± 0.5 (16)	8 ± 1 (12)	^h^ND
**9**	56 ± 0.1	8 ± 2 (7)	6 ± 0.5 (9)	8 ± 1 (7)	6 ± 0.7 (9)	5 ± 1 (11)	6 ± 0.7 (9)	7 ± 1 (8)	ND
**10**	>100	7 ± 0.8(>14)	6 ± 3(>16)	7 ± 0.7(>14)	5 ± 2(>20)	6 ± 0.5(>16)	5 ± 0.5(>20)	7 ± 1(>14)	ND
**11**	50 ± 4	5 ± 2 (10)	5 ± 0.5 (10)	5 ± 0.2 (10)	2 ± 0.7 (25)	5 ± 0.5 (10)	6 ± 0.5 (8)	4 ± 0.7 (12)	ND
**12**	73 ± 3	13 ± 2 (5)	7 ± 0.7 (10)	11 ± 2 (6)	9 ± 0.5 (8)	9 ± 0.7 (8)	7 ± 1 (7)	9 ± 2 (8)	ND
**13**	33 ± 0.1	11 ± 4 (3)	6 ± 0.9 (5)	8 ± 1.4 (4)	3 ± 1 (10)	7 ± 1 (4)	7 ± 2 (4)	8 ± 2 (4)	ND
**14**	40 ± 2	5 ± 1 (8)	7 ± 1.4 (5)	3 ± 1 (13)	7 ± 1 (13)	6 ± 0.7 (6)	6 ± 1 (6)	4 ± 0.7 (10)	ND
**15**	66 ± 3	15 ± 4 (4)	7 ± 0.1 (9)	8 ± 2 (8)	8 ± 1.5 (8)	6 ± 1 (11)	11 ± 3 (6)	8 ± 2 (8)	ND
**16**	86 ± 1	16 ± 5 (5)	7 ± 0.4 (12)	11 ± 2 (7)	10 ± 3 (8)	8 ± 0.7 (10)	13 ± 2 (6)	12 ± 2 (7)	ND
**17**	73 ± 0.1	2 ± 1 (36)	5 ± 0.7 (14)	9 ± 3 (8)	5 ± 2 (14)	4 ± 0.7 (18)	3 ± 0.5 (23)	6 ± 1 (12)	4 ± 0.5 (18)
**18**	>100	16 ± 3(>6)	14 ± 1(>5)	20 ± 4(>5)	7 ± 2(>14)	12 ± 2(>8)	16 ± 2(>6)	15 ± 3(>6)	ND
**19**	60 ± 4	11 ± 3 (5)	14 ± 3 (4)	≥ 33	7 ± 2 (8)	14 ± 1 (5)	11 ± 2 (5)	21 ± 1 (2)	12 ± 2 (5)
**20**	>100	22 ± 3.5 (4)	23 ± 1 (4)	25 ± 2(>4)	22 ± 1(>4)	20 ± 2(>5)	19 ± 1(>5)	25 ± 3(>4)	ND
**21**	>100	76	44	58	70	70	63	63	ND
**22**	>100	21(>4)	20(>5)	20(>5)	21(>4)	24(>4)	25(>4)	23(>4)	ND
**23**	36 ± 2.5	26 ± 2	23 ± 1	24	25	22	25	22	ND
**24**	>100	71 ± 6	29 ± 1	56	65	71	64	63	ND
Reference									
EFV	30 ± 5	0.002 ± 0.0003	0.008 ± 0.003	0.3 ± 0.009	3 ± 1	0.002 ± 0.0003	0.004 ± 0.05	0.007 ± 0.005	>20
AZT	≥20	0.01 ± 0.003	0.02 ± 0.001	0.01 ± 0.003	0.01 ± 0.002	0.2 ± 0.05	0.2 ± 0.07	0.01 ± 0.005	0.008 ± 0.001
SAQ	≥20	0.01 ± 0.001	0.02 ± 0.001	0.01 ± 0.002	0.01 ± 0.005	0.01 ± 0.005	0.02 ± 0.007	0.5 ± 0.1	ND

Data represent mean values + SD for three independent determinations. For values where SD is not shown, the variation among duplicate samples was less than 15%. ^a^ Compound concentration (μM) required to reduce the viability of mock-infected MT-4 cells by 50%, as determined using the MTT method. ^b^ Compound concentration (μM) required to achieve 50% protection of MT-4 and C8166 cells from HIV-1-induced cytopathogenicity, respectively, as determined by the MTT method. Data represent mean values + SD for at least three independent determinations. The Selectivity Index (SI = CC_50_/EC_50_) is shown in round brackets.^c^HIV-1: IIIB strain. ^d^NNRTI-resistant mutants: N119 (Y181C); A17 (K103N + Y181C); EFV^R^ (K103R + V179D + P225H). ^e^NRTI-resistant mutants: AZT^R^ (67 N, 70R, 215F, 219Q); MDR (74 V, 41 L, 106A, 215Y). ^f^ PRI-resistant mutant: SAQ^R^ (L10F, G48V, L90M). ^g^HIV-2 CBL-20 strain.

The comparative activities of derivative 6, EFV, and Amphotericin B (AMPH-B) against HIV-2 are summarized in Table 2. Compound **6**, similar to AMPH-B (Waheed et al., 2008; Otake et al., 1991; Pleskoff et al., 1995; Hansen et al., 1990), was equally potent against HIV-2 and HIV-1 wt, but also against variants containing mutations conferring resistance to NNRTIs (Table 1).

**Table 2:** Comparative activity of compound **6** and reference compounds against HIV-1 and HIV-2 in cell-based assays.

Compounds	HIV-1	HIV-2
	^a^EC_50_ [μM]	
**6**	8 ± 3	7 ± 0.7
AMPH-B	6 ± 3	2 ± 0.3
EFV	0.002 ± 0.0003	>20

^a^Compound concentration [μM] required to achieve 50% protection of MT-4 cells from HIV-1-induced cytopathogenicity, as determined by the MTT method. Data represent mean values + SD for at least three independent determinations.

### 3.2 Study of the mechanism of action of benzenesuphonamide derivatives

#### 3.2.1 Enzymatic assays

Compounds **6**, **7**, and **19** were selected and subjected to further evaluation, as described in our previous studies (Costi et al., 2004; Tramontano and Cheng, 1992), in enzyme assays aimed at investigating their capability to inhibit recombinant HIV-1 RT and IN. Interestingly, unlike reference drugs [EFV (IC_50_ on rRT = 0.06 μM) and the diketoacid inhibitor of rIN, L-731,988 (internal control) (IC_50_s on 3′ processing and strand transfer = 2.5 and 0.35 μM, respectively)], none of the benzenesulfonamides were active at concentrations of 30 μM (Supplementary Tables S1, S2), thus making it unlikely that RT or IN might be their target enzymes.

#### 3.2.2 Entry assay

**Figure 2:**
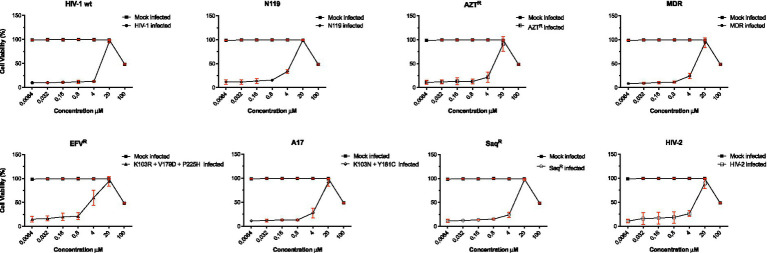
Cytotoxicity and anti-HIV-1 activity of derivative 6. To evaluate antiviral activities of compound **6** in HIV-1 wt and drug-resistant strains, several concentrations of compound **6** (100–0.0064 μM) were used to inhibit wt IIIB and clinically relevant RT-inhibitor-resistant strains (EFVR, N119, A17, AZTR, MDR, SaqR, and HIV-2). The viability of all HIV-1-infected MT-4 cells or C8166 for HIV-2 was estimated by MTT assay 4 days post-infection. The number of live cells was expressed as a percentage of mock-infected, untreated control cells. Data are expressed as the mean ± S.D. of at least three independent measurements.

The potential of compound **6** to interfere with HIV entry mechanisms is being investigated using coculture experiments between chronically HIV-1-infected H9 cells and uninfected C8166 cells (Wang et al., 2004). Compound **6** was selected as the lead candidate based on its favorable antiviral profile and cytotoxicity data, as summarized in the EC_50_ curves in Figure 2.

**Figure 3:**
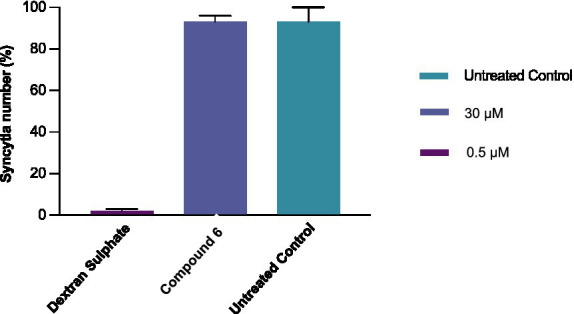
Comparative activity of compound **6** (30 μM) and dextran sulphate (0.5 μM) in preventing syncytium formation in chronically infected H9/C8166 cocultures. The H9/C8166 ratio was 1:550. The cells were incubated for 36 h at 37 °C. Data represent mean values of at least three independent determinations of syncytial numbers using an optical microscope. Bars are mean values, and whiskers are standard deviations (SD) of at least three independent experiments.

In this assay, syncytia were formed as a result of the interaction of env-encoded glycoproteins (present on the outer membrane of chronically infected H9 cells) with the CD4 of cocultured C8166 cells. In contrast to dextran sulphate, 0.5 μM, which prevented syncytia formation by 98%, 6, tested at 30 μM, did not interfere with the very early events (adsorption/attachment/fusion) in the HIV replication cycle triggered by the gp120-CD4 interaction (Figure 3) (Baba et al., 1988; Mitsuya et al., 1988).

**Figure 4:**
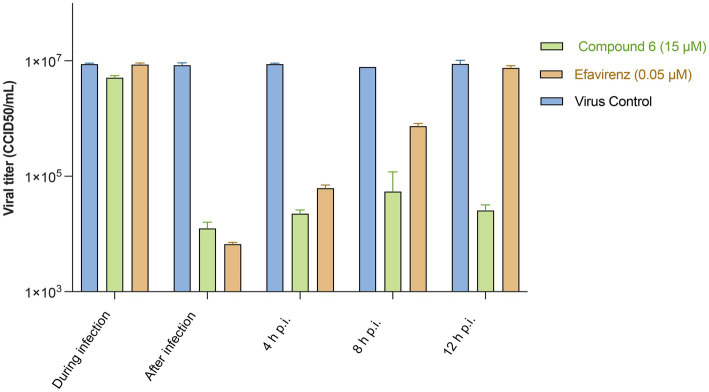
HIV-1 titres in supernatants of cultures treated with compound **6** and EFV during infection or at different times p.i. In a time-of-addition assay, MT-4 cells were inoculated with HIV-1 wt (MOI = 5), and then compound **6** (15 μM) or EFV (0.05 μM) was added at the indicated time points. Virus yields were determined using the Reed–Muench method. Bars are mean values, and whiskers are standard deviations (SD) of three independent experiments.

To identify the step(s) of the HIV-1 infection cycle targeted by compound **6**, a time-of-addition study was performed in MT-4 cells under a single round of viral replication, in comparison with EFV, a drug representative of the NNRTI antiretroviral class (Figure 4).

None of the tested compounds inhibited HIV-1 multiplication when present only during the 1-h infection period, as revealed by the viral yield. In particular, both EFV and compound **6** produced an infectious virus yield similar to that of the untreated infected controls (Figure 3), suggesting that, in addition to not affecting HIV-1 entry, compound **6** (15 μM) did not accumulate and persist in the cells. EFV, which is capable of targeting RT without prior metabolism, was still inhibitory even if added within 4 h p.i. (Figure 4), suggesting that under the experimental conditions used, reverse transcription was essentially completed by compound **6** h p.i. Interestingly, compound **6** resulted in effective prevention of virus yield (regardless of whether it was added to the cultures immediately at the end of the infection period or any time) up to 12 h later. These data suggest that compound **6** target events can occur both early and late in the HIV infection cycle.

#### 3.2.3 Benzenesuphonamide derivatives’ direct infectivity inactivation of virions

Thus, to investigate the potential virucidal activity of our candidate compound, compound **6** was incubated for 2 h at 37 °C with a high-titre HIV-1 stock solution, whose residual infectivity was then determined using the Reed–Muench endpoint titration method.

**Figure 5:**
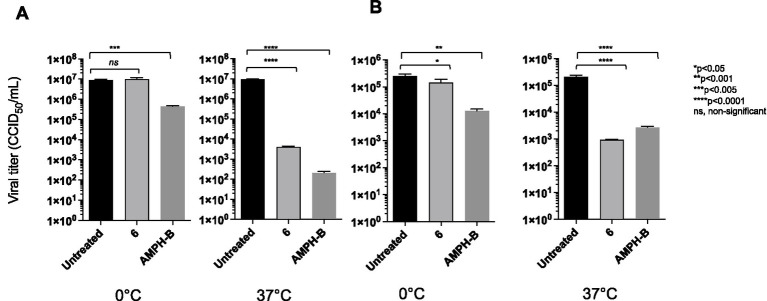
Comparative virucidal effects of the derivative against HIV-1 **(A)** and HIV-2 **(B)**. For virucidal activity evaluation, virions were treated with compound **6** at either 0 °C or 37 °C for 1 h. Black columns for viral titre for untreated viral solution and light grey columns for viral solution treated with derivative 6. Dark-grey columns viral solution treated with AMPH-B. Viral yields were determined using the Reed–Muench method (CCID_50_/ml). ANOVA post-tests were performed for all groups. ****p < 0.0001, ***p < 0.001, **p < 0.005, *ns*, non-significant.

**Figure 6:**
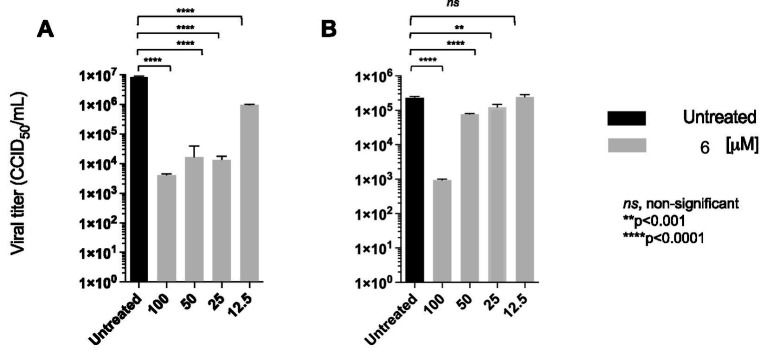
Compound **6** concentration-dependent inactivation of cell-free infectious HIV-1 **(A)** and HIV-2 **(B)**. Viral particles were directly treated with different concentrations (100–12.5 μM) of compound **6** at 37 °C. Quantification of viral titres was performed, and results for each treatment group are presented CCID_50_/ml. ANOVA post-tests were performed on all groups. **** p < 0.0001 (higher concentrations of 6), compared to the HIV-1 and HIV-2 control group (untreated).

Amphotericin B, a cholesterol-depleting compound capable of inhibiting HIV-1 infectivity, was used as a reference drug because of its direct virucidal properties (Waheed et al., 2008). When used at concentrations approximately 10- and 15-fold higher than the respective EC_50_s, both compound **6** (80 μM) and AMPH-B (100 μM) were capable of inactivating HIV-1 infectivity by more than 3 log10 (Figure 5). Interestingly, when the incubation was carried out at 0 °C, compound **6** failed to affect HIV-1 infectivity, while AMPH-B was still able to reduce it by a high percentage. When the same experiment was carried out with HIV-2, both compound **6** and AMPH-B inactivated virion infectivity by approximately 2 log10 (Figure 5). Again, if the incubation was carried out at 0 °C, compound **6** failed to significantly affect HIV-2 infectivity, whereas AMPH-B reduced it by more than 1 log10 (Figure 5). Infectivity inactivation of both HIV-1 and HIV-2 was determined by exposing cell-free stock solutions to serial dilutions of 6. As shown in Figure 6, concentrations of 100 and 50 μM strongly inactivated the infectivity of both HIV types in a concentration-dependent manner; conversely, lower concentrations (25 and 12.5 μM) had only marginal or no effects on HIV infectivity.

#### 3.2.4 *In vitro* selection of a resistant mutant to compound **6**

**Figure 7:**
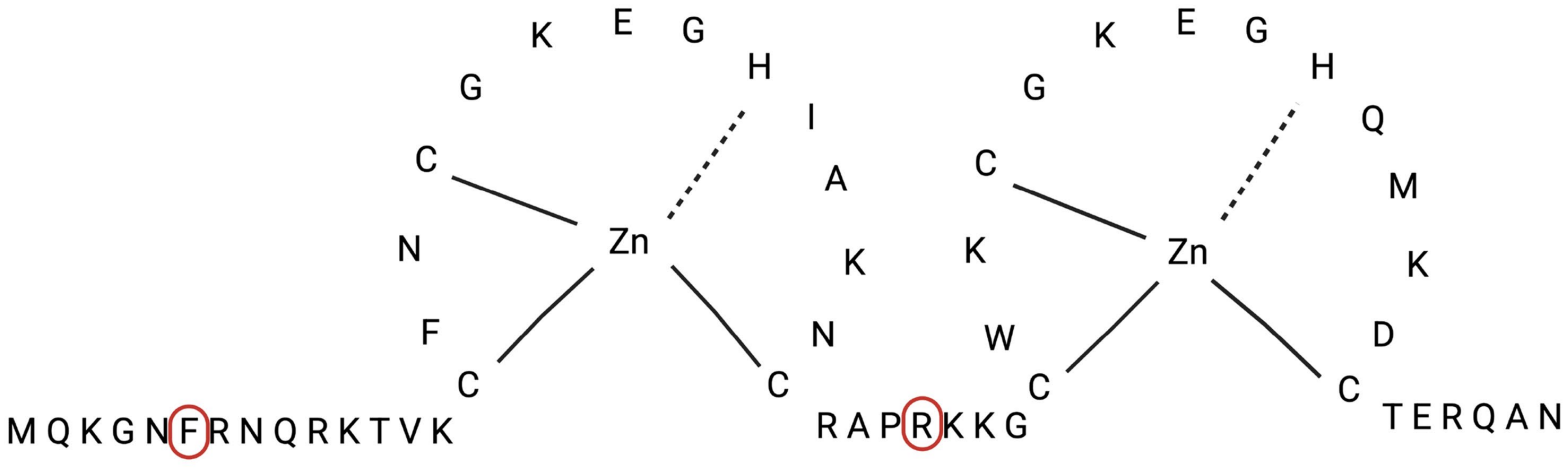
Amino acid sequence of NCp7 protein, with localization of amino acids (Phe-6 and Arg-32) involved in mutations of HIV-1^**6**^ strain.

Mutant viruses resistant to compound **6** were selected by serial passages of HIV-1_IIIB_ in the presence of stepwise doubling of drug concentrations, starting from a cell culture infected with an m.o.i. of 0.01, and treated with a drug concentration equal to the EC_50_ of the drug. The amount of virus obtained after each passage was usually sufficient to continue infection of the next cell culture, which, after infection and washing of the unadsorbed inoculum, was incubated with double the amount of the selected drug. However, in the case of 6, we had to keep the drug concentrations unchanged at passage 3 in order to obtain enough infectious virus to propagate through the next passages. With this single restriction, compound **6**-resistant mutants (HIV-1^6^) were grown to drug concentrations 16 times higher (16x) than the EC_50_. The selected virus population was then subjected to RNA extraction, RT-PCR, and sequencing of the *gag*, *pol*, and *env* genes to identify the mutation pattern responsible for drug resistance. Comparative genomic analysis of wild-type HIV and HIV-1^6^ mutants showed that neither had mutations in the RT and PR genes. The same was true for the *env* gene. On the contrary, two mutations, F6C and R32K, were selected in the NCp7 sequence of the *gag* gene (Figure 7). Since the presence of two well-conserved zinc-finger domains of the Cys-X2-Cys-X4-His-X4-C (CCHC) type is crucial for the activity of NCp7, it is reasonable to foresee that compound **6** binds to this domain.

## 4 Discussion

Novel synthetic 1,2-benzisothiazol-3(*2H*)-one benzenesulfonamides with a wide spectrum of antiretroviral activity against HIV-1, HIV-2, and HIV-1 variants carrying clinically relevant mutations conferring resistance to NNRTIs, NRTIs, and PRIs are described. They neither interfere with the gp120-CD4 interaction leading to HIV entry and syncytium formation nor inhibit RT or IN in enzyme assays. Nevertheless, they affect events occurring in both early and late stages of the HIV multiplication cycle and inactivate the infectivity of cell-free HIV virions in a concentration-dependent manner.

Two mutations, F6C and R32K, were detected in the *gag* region coding for NC proteins in mutants selected for resistance to compound **6**. Therefore, since mutations evolve to counteract the binding of the drug to its target, it is reasonable to suggest that in *wt* viruses, NC proteins are the target of 6. The fact that NC proteins are involved in both early reverse transcription (Carteau et al., 1997; Zybarth and Carter, 1995), integration (Aldovini and Young, 1990), late protease processing (Thomas and Gorelick, 2008), and packaging of viral genomic RNA (Arner et al., 1996) stages of the HIV-1 replication cycle, in addition to being structural components of mature virions, is consistent with both inhibition of HIV multiplication in infected cells and direct inactivation of virion infectivity.

NC retrovirus proteins are synthesized as functional domains of the Gag precursor. In HIV-1, mature NC is generated late in the assembly before virus budding takes place by a cascade of PR-mediated cleavages of Pr55^Gag^. End-products are matrix (p17^MA^), capsid (p24^CA^), and nucleocapsid (NCp7) proteins, p6, and smaller peptides (Clerici et al., 2007).

NC proteins, whether as domains of Pr55^Gag^, intermediate cleavage products (p15^NC^ and p9^NC^), or mature NCp7, are characterized by two zinc finger sequences, C-X_2_-C-X_4_-H-X_4_-C (CCHC), which are highly conserved among retroviruses. Zinc finger sequences are linked by a central spacer, RAPRKKG, and flanked by the NH_2_-terminal (MQRGNFRNQRKIVK) and COOH-terminal (TERQAN) regions, which are highly conserved within a particular retrovirus species. As a matter of fact, HIV-1 NC proteins function as nucleic acid chaperones; in fact, they destabilize nucleic acid helices (through zinc fingers) and elicit nucleic acid aggregation (through basic residues). NC proteins are absolutely required for viral replication, and genetic analyses have proven that many, even minor, alterations can affect virus assembly by disrupting genomic RNA (gRNA) recognition and packaging or formation of infectious particles (Clerici et al., 2007), consistent with the results described in this paper. Evidence for the binding of isothiazolones to cysteine (Cys) has been claimed (Arner et al., 1996), as well as for the liability of the N-S bond that involves a nucleophilic attack by the sulfur atom of Cys at the sulfur of the isothiazolone moiety, followed by the cleavage of the N-S bond (Clerici et al., 2007). When binding to cysteine occurs in the HIV-1 NCp7 Zn-finger domain, zinc extrusion (with subsequent denaturation of the viral protein) occurs for several thio-compounds bearing proper electrophilic groups (Musah, 2004; Schito et al., 2006; Basrur et al., 2000; Ranganathan et al., 2002; Topol et al., 2001; Tummino et al., 1997; Loo et al., 1996).

While our study provides valuable initial insights, we are aware that several significant limitations prevent definitive conclusions about the resistance mechanism and target identification. Genetic analysis was constrained to only three genomic regions (*gag, pol*, and *env*), which represent narrow targets that may miss critical resistance-conferring mutations in other viral genes. This limited sequencing approach leaves substantial gaps in our understanding of the complete mutational landscape associated with the development of resistance.

This approach makes it unclear whether the identified F6C and R32K substitutions occur within the same viral genome or whether they are independent mutations in different viral particles within the population.

Without experimental validation, the functional significance of the F6C and R32K mutations remains speculative. Site-directed mutagenesis studies are essential to definitively establish the causal relationship between these specific substitutions and the development of resistance. Without this, we cannot rule out the possibility that other undetected mutations are primarily responsible for resistance.

This study also presents additional constraints: the observed resistance level of 16 × EC₅₀, while significant, represents a relatively modest fold-change, and the lack of cross-resistance profiling against related nucleocapsid inhibitors further restricts our ability to evaluate broader therapeutic implications.

The novel finding of this study is the establishment of the ability of compound **6** to induce F6C and R32K mutations that could be responsible for drug resistance by inducing a conformational change in the NCp7 structure, preventing its interaction with compound **6**. This is consistent with the activity shown by compound **6** against HIV-1 laboratory strains carrying clinically relevant NNRTI mutations and HIV-2, which occurs at concentrations (7–8 μM) similar to those active against the *wt* strain.

These observations lead us to suggest an inhibitory mechanism involving the NCp7 protein as a target. Further analysis of site-directed mutagenesis will be performed to (i) confirm the correlation between the observed mutations and the degree of resistance to compound **6** and (ii) investigate the contribution of each mutation to resistance.

## 5 Conclusion

A new class of molecules with an unusual spectrum of antiretroviral activity has been identified. The experiments clearly show that the selected compound **6** does not inhibit HIV-1 reverse transcriptase, integrase, or viral attachment and fusion to host cells. Notably, compound **6** was equally potent and selective against HIV-2 and HIV-1 variants, with mutations conferring resistance to NNRTIs. In a comparative assay for virucidal activity, AMPH-B and compound **6** turned out to be active in inactivating the HIV-1 infectivity of an HIV-1 stock solution, showing virucidal activity, the former at both 0 °C and 37 °C, and the latter only after incubation at 37 °C. Consistent with this, genome analysis of 6-resistant mutants (selected up to 16-fold EC_50_) shows that no significant mutation was selected in the RT and protease genes, as well as in the env gene, while two mutations (F6C and R32K) were selected in the NCp7 gene of the gag polyprotein, which is involved in genomic RNA packaging and virus particle morphogenesis, suggesting that NCp7 may be the target of the inhibitory action of compound **6**.

Although we recognize that compound **6** is not very potent, its moderate selectivity index suggests that the therapeutic window may be narrow; however, this does not rule out further development. Although we acknowledge this as a limitation, it also presents an opportunity for targeted optimization in future studies. This compound has an unusual spectrum of activity and an interesting mode of action. It is an antiviral that targets a fundamental part of the viral structure rather than an enzyme; therefore, it merits further investigation.

## Data Availability

The original contributions presented in the study are included in the article/Supplementary material, further inquiries can be directed to the corresponding author/s.
