# Supplementary material for: HIV nucleocapsid proteins as targets for novel 1,2-benzisothiazol-3(2H)-one benzenesulfonamides: synthesis and antiretroviral activity
Source: Front Microbiol. 2025 Nov 5;16:1664231. doi: 10.3389/fmicb.2025.1664231 (PMC12627007; doi:10.3389/fmicb.2025.1664231)
Supplement: Supplementary file 1 [file Supplementary_file_1.doc]

# Supporting Information

**HIV nucleocapsid proteins as targets for novel 1,2-benzisothiazol-3(2H)-one benzenesulfonamides: Synthesis and antiretroviral activity.**

Roberta Loddo^1^a, Matteo Incerti^1^*b, Valeria Manca^a^, Vanessa Palmas^a^, Marta Cogoni^a^, Rebecca Piras^a^, Luca Virdis^a^, Elena Tamburinia, Elda Favarib, Paolo La Collaa, and Giuseppina Sanna*a

aDipartimento di Scienze Biomediche, Sezione di Microbiologia e Virologia; Università degli Studi di Cagliari; Monserrato, (CA), 09042; Italy.

bDipartimento di Scienze degli Alimenti e del Farmaco; Università degli Studi di Parma; Parma, (PR), 43124; Italy.

#### Contents:

Primers and parameters used in the PCR reactions S1

Tables S1-S2

**Primers and parameters used in the PCR reactions.** The primer used in the RT reaction were OUTR 5’-CATTGCTCTCCAATTACTGTGATATTTCTCATG-3’, that binds about 400bp downstream the RT gene, and ENVR6 5’-CTGCTGTGTTGCTACTTGTGATTG-3’, that binds about 160bp downstream the *env* gene. cDNAs were amplified by PCR using Pfx polymerase (INVITROGEN), following the manufacturer’s protocol. The regions containing the *protease* and *RT* genes were amplified by PCR reactions carried out with the primers INF (5’-TGAAAGATTGTACTGAGAGACAGG-3’) and INR (5’-TCTATTCCATCTAAAAATAGTATT TTCCTGATTCC-3’), respectively. PCR consisted of: initial denaturation of 3 min; 30 cycles of denaturation at 94 °C for 30s, annealing at 52 °C for 30s and extension at 68 °C for 2.5 min; final extension at 68 °C for 5 min. The *gag* region was amplified by two different PCR reactions, carried out with the primers GF1 (5’-GCGACTGGTGAGTACGCCAAAAAT-3’) and GR2 (5’-CAGCCAAAACTCTTGCCTTATGGC-3’) and with the primers GF2 (5’-TCAGCCCAGAAGT GATACCCATGT-3’) and GR1 (5’-GGTTTCCATCTTCCTGGCAAACTC-3’), respectively, in a similar PCR amplification of the above described, with the only differences of the annealing at 51.5 °C and the extension at 68 °C for 1.5 min. The *env* region was amplified by two different PCR reactions, carried out with the primers EF1 (5’-CTCAAGGCAGTCAGACTCATCAAG-3’) and ER2 (5’-TTGGTGGGTGCTACTCCTAATGGT-3’) and with the primers EF2 (5’-GAGGACCAGGGAGAGCATTTGTTA-3’) and ER1 (5’-AGCAGGCCATCCAATCACACTA CT-3’), respectively, under PCR amplification steps similar to the first ones described, with the only differences of annealing at 54 °C and extension at 68 °C for 1.5 min.

**Table S1**. Anti-RT activities of selected title compounds in enzyme assays.

| **Compds** | **^a^IC_50_ [μM]** |
| --- | --- |
| **6** | >30 |
| **7** | >30 |
| **19** | >30 |
| **EFV** | 0.06 |
| **NVP** | 4.6 |

^a^Compound concentration [μM] required to inhibit the HIV-1 virion-associated RT activity by 50%. Data represent mean values for three independent determinations. Variation among duplicate samples was less than 15%. Very similar results were also obtained with the recombinant RT.

**Table S2**. Anti-rIN activity of selected title compounds in enzyme assays.

|  | ^a^**IC_50_** **[μM]** | |
| --- | --- | --- |
| **Compds** | **3’-Processing** | **Strand-transfer** |
| **6** | >30 | >30 |
| **7** | >30 | >30 |
| **19** | >30 | >30 |
| **L 731,988** | 2.5 | 0.3 |

^a^Compound concentration (μM) required to reduce the amount of 3’-end-labeled duplex of 21/19mers substrates, in 3’-processing and strand-transfer reactions catalyzed by the recombinant HIV-1 integrase, by 50%. Data represent mean values for three independent determinations. Variation among duplicate samples was less than 15%.
